# Minimally Invasive Access Cavities: A Benefit/Risk Analysis

**DOI:** 10.3390/jcm14072476

**Published:** 2025-04-04

**Authors:** Marie Sevin, Edouard Orio, Anne-Margaux Collignon

**Affiliations:** 1 Université Paris Cité and Sorbonne Paris Nord, F-92120 Montrouge, France; marie.sevin@aphp.fr; 2 Oral Medicine Department, DMU ESPRIT, Louis Mourier Hospital, AP-HP, F-92700 Colombes, France; edouard.orio@aphp.fr

**Keywords:** endodontic treatment, access cavities, minimally invasive

## Abstract

**Background/Objectives:** Contemporary dentistry aims to preserve healthy tissues and perform minimally invasive procedures. The availability of ever-improving equipment allows practitioners to follow this conceptual innovation. This approach is also used in endodontics, as new types of access cavities seem to be gaining popularity, allegedly reducing tissue destruction and loss of mechanical resistance of the treated teeth. **Methods:** We performed a comprehensive review of the available literature on the subject, focusing on in vitro studies accessible through major search engines and limiting the search to English-language articles published between 2010 and 2024. **Results:** Our analysis showed that the realization of reduced access cavities seems to preserve the mechanical resistance of the treated teeth, may compromise disinfection, and respects the original root canal path and the quality of obturation. In addition, these procedures appear to increase instrument deformation, fracture susceptibility, and treatment time: Mini-invasive cavities have many limitations and should only be used in situations where there is a high likelihood of success, where there are few difficulties and where sufficient material is available. However, with appropriate case selection, these new approaches can be used and should improve the prognosis of endodontically treated teeth.

## 1. Introduction

Endodontic treatments are common and reproducible [1]. They aim to transform a pathological tooth into an asymptomatic and functional unit within the dental arch and achieve both biological and functional objectives [2].

**Figure 1:**
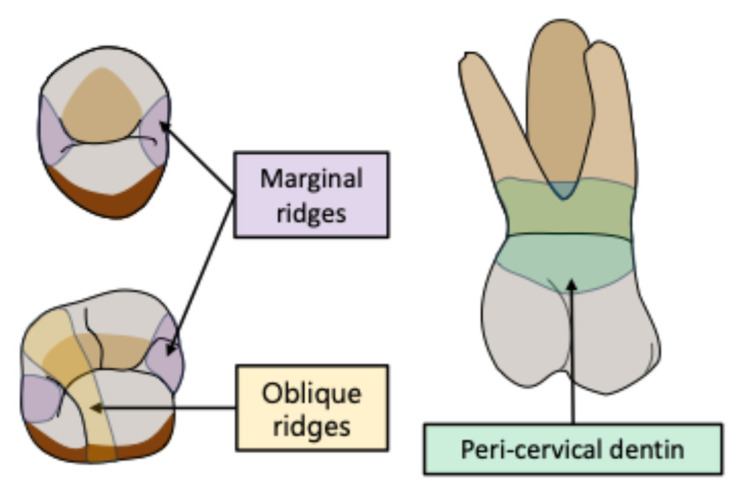
Key structures for mechanical resistance in teeth.

It is known that root canal-treated teeth are susceptible to fracture due to loss of mechanical properties as a result of tooth deterioration (caries/traumatic injury), restorative preparation and endodontic procedures [3]. In fact, the loss of certain structures, such as marginal ridges, oblique ridges and peri-cervical dentin, results in reduced resistance to cuspal flexion (Figure 1) [4,5,6,7].

In fact, maximal preservation of the non-compromised tissues and mechanical strength of the tooth undergoing endodontic treatment are one of the goals in this field [1,8]. To achieve this objective, more tissue-preserving practices emerged over the past decade [9,10]. Silva et al. proposes classifications and prospects for these cavities [11].

A recent study shows a growing interest in minimally invasive access cavities (MIACs) [12]. It reveals that 43% of participants, all members of the American Association of Endodontists (AAE), report utilizing conservative access cavities, while 57% use traditional cavities. When comparing the ages of practitioners, 63% of those under 35 years old report using conservative access cavities, compared to 44% of those over 50 years old. It appears to be a widespread practice, especially among young practitioners.

To support these new techniques, the European Society of Endodontology (ESE) recommends that the access cavity should allow the preservation of as much healthy tissue as possible [8]. Then, in 2020, the AAEs supported the contribution of flexible and fatigue-resistant instruments which allow the size of the access cavity to be reduced and eliminate the need for divergent walls [1].

Finally, the fundamental concepts of these MIACs are as follows [13]:

- Preservation of a more or less significant portion of the pulp chamber roof.
- Formation of soffits (parts of the pulp chamber roof left intact, resulting in the presence of undercut areas in the pulp chamber).
- Preservation of the peri-cervical dentin and of the tooth’s natural ferrule effect.
- Customized access cavity design for the treated tooth.

They symbolize a shift from a model centered on the needs of the operator to one centered on the benefits to the tooth [10]. However, the implementation of tissue-preserving therapies requires a combination of a favorable risk–benefit ratio, procedural technologies and adaptation of the clinician’s technical skills [14].

We attempted to evaluate the clinical relevance of these practices by answering the following questions:

- Is there an improvement in mechanical properties when preparing MIACs compared to traditional ones? Such an improvement should correspond to the main objective of this kind of cavity.
- Does the MIAC approach compromise the quality of the endodontic treatment (including debridement, disinfection, obturation and procedural errors)?
- Finally, is it possible to meet the demands of high-quality endodontic treatment with a reduced access cavity?

The aim of this article was to provide a current review of application of “minimal access cavities” in endodontic treatment, in dental practice.

## 2. Material and Method

An in-depth review was carried out of the available literature on the subject, focusing on English-language articles that could be accessed via the main search engines (PubMed, Google Scholar, ScienceDirect and Cochrane) and restricting the search to articles published between 2010 (the first articles discussing the concept) and 2024. The terms used were “pulp cavity” (MeSH), “endodontics” (MeSH), “root canal preparation” (MeSH), “conservative treatment” (MeSH), “ninja”, “contracted” and “conservative”. Articles were screened based on their relevance to “a Minimally invasive access cavities for endodontic treatment”, considering their titles. Only original articles published in prominent indexed journals in the dental sector, with or without an impact factor, were eligible for consideration.

**Figure 2:**
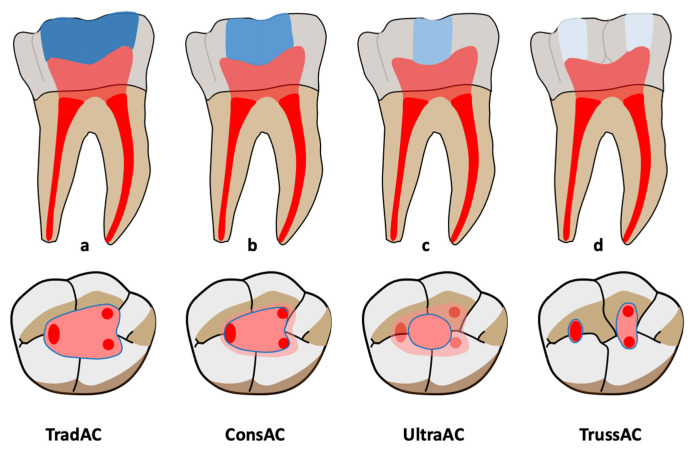
Different types of access cavities for posterior teeth. (**a**) Traditional access cavity (TradAC); (**b**) Conservative access cavity (ConsAC); (**c**) Ultra-conservative access cavity (UltraAC); (**d**) Truss access cavity (TrussAC). Light red for coronal pulp. Dark red for radicular pulp. Gradient of blue migrates from more invasive cavities to less invasive cavities.

Exclusion criteria were studies conducted on resin teeth, opinion articles, case reports, systematic reviews and meta-analyses. To be selected, the studies were required to compare traditional access cavities with at least one type of MIAC described here [15,16] (Figure 2):

- Traditional access cavities (TradACs): Prepared using standardized occlusal landmarks. Complete removal of the pulp chamber roof, followed by a straight-line access to the canal orifices with slightly divergent walls, allowing a convenient form to be obtained. All orifices can be seen simultaneously from the entry point of the access cavity. Pre-enlargement of the canal openings is recommended.
- Conservative access cavities (ConsACs): convergent cavity walls; a preserved pulp chamber roof which leads to the formation of soffits.
- Ultra-conservative access cavities (UltraACs; ninja ACs): A single-entry point is created. No direct visual access to the canals is obtained.
- Truss access cavities (TrussACs): multiple entry points are created for each canal. The preserved pulp chamber roof acts like a load-bearing beam.

The results presented in this article were extrapolated from this literature search, with reference to the authors’ clinical knowledge.

## 3. Results

Following this search, 24 articles were identified, including 23 in vitro studies and a single in vivo study [14,17,18,19,20,21,22,23,24,25,26,27,28,29,30,31,32,33,34,35,36,37,38,39], and seven relevant criteria have been highlighted (Table 1): mechanical resistance, canal orifice detection, instrumentation quality, canal deviation, instrument-related constraints, obturation and time.

### 3.1. Mechanical Resistance

For unrestored teeth, Sabeti et al. found no significant difference between conservative and traditional access cavities in maxillary molars [35]. Krishan et al. reported a significant increase in fracture resistance between conservative access cavities compared to TradACs in premolars and molars, but no difference was observed in incisors [25].

Regarding the in vitro studies comparing different access cavities on intact teeth post-endodontically restored with direct restoration techniques, no significant difference was described between traditional, conservative, ultra-conservative and Truss access cavities on molars, premolars and incisors [14,20,22,27,31,33,36,37]. Only Plotino et al. showed a significant decrease in the fracture resistance of molars and premolars with TradACs compared to conservative and ultra-conservative access cavities, and no significant difference was found between the latter two [32].

Abou-Elnaga et al. demonstrated a significant improvement in the mechanical resistance of degraded mandibular molars (with loss of two or three walls) when TrussACs were utilized compared to TradACs [17]. However, other studies have reported no significant difference in fracture resistance between various access cavity designs, including TradACs, ConsACs, and TrussACs [22], or between TradACs and consACs [30] if the tooth is degraded.

### 3.2. Canal Orifice Detection

MIACs affect canal orifice detection due to a limited field of view and reduced brightness due to a smaller occlusal opening. There is a real difference in canal detection between traditional and conservative cavities, but the use of microscopes and ultrasound improved canal orifice detection for second mesio-buccal (MB2) canals in maxillary molars when a conservative access cavity is used [33].

### 3.3. Instrumentation Quality

Regardless of the instrument used, the proportion of uninstrumented areas did not differ significantly between traditional and conservative access cavities for incisors [25,34,39], maxillary premolars [37] and mandibular premolars or maxillary molars [14,25,33]. A significant number of untouched walls were observed in mandibular molars [20,24,27] and bifurcated maxillary premolars [31]. However, the analyzed studies highlight a greater proportion of residual tissues in the pulp chamber of teeth with ultra-conservative cavities [37]. Analyses of histological sections reflected similar results for TrussACs compared to TradACs, but did not show any difference in terms of residual pulp tissue in the canals and isthmuses of mandibular molars [29].

In terms of debris accumulation due to instrumentation, there was no significant difference between the different access cavities tested [27,33,34]. However, Silva et al. showed an increase in dentin debris accumulation in UltraACs [37].

Regarding the reduction in bacterial load, Barbosa et al. found no difference in bacterial count between traditional cavities, consACs and TrussACs when cultured bacteria were collected before and after shaping [20]. However, Vieira et al. using qPCR analysis, compared TradACs and consACs and identified an increased bacterial load for MIACs [39]. Al-Ani’s in vivo study suggested no difference in bacteria reduction before and after shaping when comparing TradACs and ConsACs [18].

### 3.4. Canal Deviation

Five studies did not reveal an alteration in the canal path when TradACs and ConsACs were confronted in terms of canal deviation and canal centering after instrumentation [20,25,26,28,34]. Marchesan et al. noted no significant difference in the position of the curvature, the degree of the angle and its radius [28].

However, four others showed significantly more canal transport from the center of the root for conservative or ultra-conservative access cavities [19,27,31,33].

No difference in residual dentin thickness was found between traditional and Truss access cavities [25] or between TradACs and ConsACs [24].

There is no standardization of the teeth or instrumentation system used between different studies.

### 3.5. Instruments’ Constraints

Studies showed little or no instrumental separation regardless of the instruments system used [14,19,20,27,28,33,38]. However, Silva et al. reported evidence of ductile fractures or a significant reduction in time to fracture for the instruments used in the UltraACs group if they were used until they fractured [38].

### 3.6. Obturation

A significant increase in the proportion of voids within the canal filling material was found in ConsACs [34] or UltraACs [27] compared to TradACs. Some authors did not find a significant difference between ConsACs, TradACs and TrussACs [20] or between UltraACs and TradACs [37].

In terms of coronal filling, more voids were found in ConsACs compared to TradACs [31]. A greater amount of persistent filling material in the pulp chamber was also observed in ConsACs [20,27,31,37]. Only Rover et al. found no difference in the persistence of filling material in the pulp chamber after cleaning with non-mechanized endodontic instruments [34].

### 3.7. Time

All the articles analyzed revealed a longer treatment time when a minimally invasive approach was used, regardless of the shaping system and tooth [19,26,27,28,37]. The treatment time could be multiplied by 2.5 when using MIACs [28].

## 4. Discussion

The aim of this work is to determine whether it is reasonably possible to recommend minimally invasive access cavities, and in which situations they have a favorable risk–benefit ratio. The analyzed studies were conducted on different types of teeth, with different type of access cavities and with different instruments systems, but we want to bring to light clinical relevance from mostly in vitro studies.

MIACs are designed to enhance the mechanical resistance of the tooth while minimizing unnecessary tissue removal. Studies on undamaged teeth comparing traditional access cavities and conservative access cavities restored using a direct composite-based method showed that this assumption is false for all types of teeth [14,20,22,27,31,33,34,37]. However, it is rare that teeth needing root canal treatment are totally undamaged. Studies using teeth with two or three missing walls are more realistic and present discrepancies in their results [17,22,30]. Taken one by one, such studies are inconclusive. But if we confront our understanding of our selected studies based on available meta-analyses, a significant increase in mechanical strength for teeth can be observed for conservative and Truss access cavities when all marginal ridges are preserved, but no difference is identified when at least one of these marginal ridges is lost [40,41,42].

Furthermore, to mimic more clinical conditions, chewing forces and cyclic fatigue resistance must be evaluated. Two studies simulated 1 year and 4 years of in vitro masticatory function before conducting fracture tests. These studies do not demonstrate significant differences between the different access cavities tested [14,27].

Missed canals are a critical clinical consideration, as they are a well-recognized cause of peri-apical disease development and endodontic failure [43,44]. They are more commonly associated with MIACs compared to traditional access cavities, as the smaller cavity size restricts light penetration and limits the field of vision [4,40,41,45]. This issue can be effectively addressed with the use of microscopes and ultrasonic instruments by the operator [40,41].

Periapical health is related to the amount of bacteria that remain in endodontic canals after root canal preparation [46]. Cleaning and shaping reduce the number of bacteria to below the threshold required for good periapical health. Shaping has two objectives in this context: it allows the irrigation solution to penetrate deep into the canal and disorganizes the biofilm present on the root canal walls. Cleaning is difficult to investigate in vitro because teeth are sterilized and the complexity of exchanges between microbial and the host response cannot be simulated. This is why authors analyze the proportion of untouched walls [14,20,21,25,27,31,33,34,37,39] and the amount of remaining debris [27,33,34,37] that can lead to bacteria development or a decrease in the number of bacteria after cleaning and shaping [20,39]. The heterogeneous results show a tendency towards deterioration in cleaning and shaping, which is confirmed by Shroff’s meta-analysis [47] and is also confirmed by Saeed in a systematic review [4].

The analyzed studies call for precautions and emphasize the importance of mechanized debridement of the pulp chamber for MIACs [20,27,31,37]. These recommendations are supported by Ballester’s meta-analysis [40].

One of the rules for preparation is to maintain the original route of the canal. Any deviation can conduct to stripping, perforation or blockage. There is a clear trend that MIACs could alter the canal deviation [20,23,26]. In addition, instruments show more signs of deterioration when they are used in conservative access cavities than in traditional access cavities [38]. A reduction in the size of the access cavity can potentially impact the mechanical behavior of shaping instruments due to the increased coronal stresses applied to them. For this both reasons, more flexible instruments should be favored [41]. In practice, even with more flexible heat-treated instruments, it seems riskier to use conservative access cavities.

For obturation, if half of the studies do not reveal more voids in root canal filling in teeth treated with MIACs compared to traditional cavities [27,34], half show the opposite [20,38]. Warm lateral condensation of the gutta-percha does not seem suitable for conservative access cavities due to the difficulty of inserting heated pluggers through the occlusal access point. Cold-lateral condensation using bioceramic cements appears more appropriate in this context, although this method has not yet been evaluated in the included studies. Interestingly, most of the studies report persistent filling material under soffits [20,27,31,37]. This can impair the coronal bounding, leading to caries and, eventually, to root treatment contamination.

The last criterion analyzed is the time of the treatment. It can be a reason for postponing obturation for patients who find it difficult to keep their mouths open, for example, particularly in mandibular molars [4]. This is the case for children, for elderly patients or patients with physical conditions (pregnant woman, people with back pain, etc.). This criterion cannot directly lead to a reduction in the success rate of root canal treatment [48]. MIACs substantially increase treatment time. As such, they cannot be recommended for all patients.

### 4.1. Decision-Making Process

When a new technique emerges, we have to ask ourselves whether it can be used safely on all types of teeth and patients. This will result in a decision tree to answer the following simple question: when can I use minimally invasive access cavities?

#### 4.1.1. Treatment Prognosis

Ng et al. suggests a higher risk of failure if the concerned tooth presents a preoperative periapical lesion [49]. As these are mostly identified via in vivo studies, it is often considered to be too early to carry out a minimally invasive treatment in these situations. Otherwise, some patients are less likely to heal due to a fragile medical context compromising the success rate, and treatment failure could induce a persistent infection [50,51]. It does not seem that this treatment can be safely administered in patients with infectious risks or when a periapical lesion is present.

#### 4.1.2. Technical Equipment

Our results highlight various elements that could compromise the treatment quality in the case of MIACs (undetected canals, remaining coronal pulp leading to lack of sealing, poor coronal and canal obturation, risk of procedural errors, etc.). Dental practitioners who want to utilize this new type of cavity must have technical equipment such as flexible nickel–titanium instruments, ultrasonic troughing and an operating microscope and be trained to use them [40,41]. In addition, poorly accessible cavities make it impossible to analyze the shape of the pulp chamber and the color of the dentin, and the low light level forces the practitioner to use magnification and an ultrasonic instrumentation to find all the canals and properly remove residual cement debris in the chamber [47]. Traditional cavities are preferred in cases where one of these instruments or techniques is not used.

#### 4.1.3. Difficulty

According to Ng et al., there are 11 clinical prognostic factors, including the presence of a periapical lesion associated with a pulp necrosis, or of a sinus tract [49]. The other factors relate to the quality of the root canal treatment itself (patency achievement, extension of the cleaning as close as possible to the apex, use of EDTA and NaOCl for final irrigation, no use of chlorhexidine, absence of perforation or overextension, or presence of a satisfactory coronal restoration).

Using a MIAC complicates each step of the root canal treatment as we described previously, which adds to the initial difficulty and increases the risk of procedural errors [40]. It is therefore necessary to limit their use to straightforward cases. We can help ourselves in the decision-making process by using a form designed to estimate the difficulty of an endodontic treatment, which is created by the AAE enabling treatments to be differentiated according to their difficulty [1].

This tool is not a substitute for the clinician’s own assessment, but it does help to rationalize their thinking. However, a per operation return to traditional access is possible when the minimally invasive cavity does not safely achieve the treatment objectives.

#### 4.1.4. Interest in Tooth Restoration

It has been demonstrated by Reeh et al. that the presence of an occlusal cavity leads to a 20% loss of rigidity, 46% loss for an occluso–mesial or occluso–distal cavity, and 63% loss for a mesio–occluso–distal cavity [45]. The authors also noted that the preparation of an access cavity causes only a 5% loss of rigidity. In a clinical situation, one should consider whether creating a MIAC would allow a less destructive technique for restoration and whether such an access cavity would improve the prognosis of the restoration.

In the case of a moderate substance loss, these techniques may be relevant if they avoid the need for cusp coverage by reducing the volume of removed tissue and enlarging the surface for restoration bonding. Concerning anterior teeth, the application of MIACs seems less suitable.

#### 4.1.5. CariesACs or RestoACs

Caries-driven access cavities/restoration-driven access cavities (CariesACs/RestoACs) are access cavities made through an initial restoration or by eliminating only the infected tissues [52].

**Figure 3:**
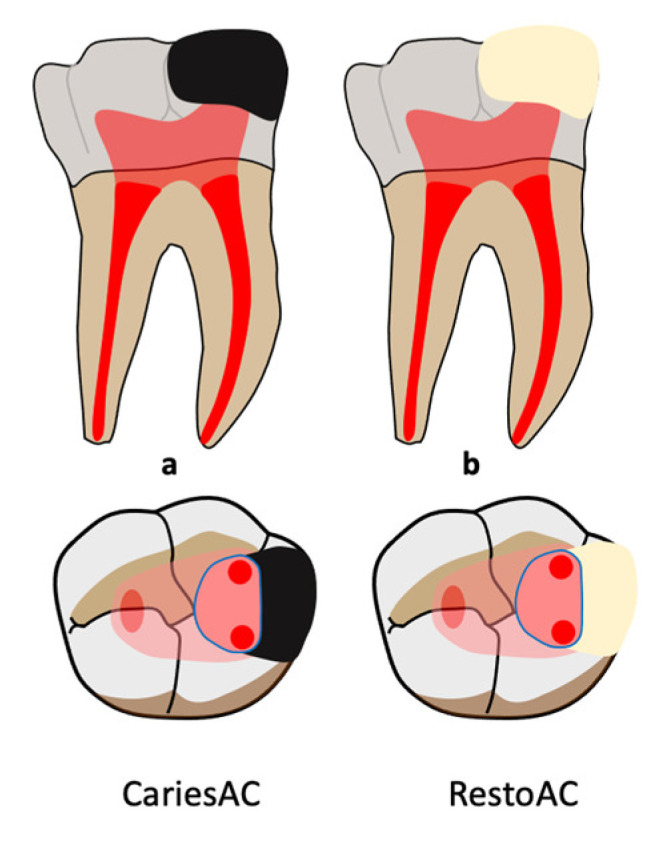
Completion of MIACs classification. (**a**) Caries-driven access cavity (CariesAC) (in black); (**b**) restorative-driven access cavity (RestoAC) (in beige). Light red for coronal pulp. Dark red for radicular pulp.

When carious lesions or restorations are present on a tooth for which endodontic treatment is indicated, it may be relevant to access the canals by eliminating only the infected tissues or the restoration (CariesAC and RestoAC) (Figure 3). This allows key structures to be maintained, but it also complicates root entries access. The preexisting restorative must be retained if it is satisfactory [40].

### 4.2. Synthesis and Proposed Decision Tree

Precise recommendations cannot be given due to the large number of parameters to be considered; each case must be analyzed individually. This decision-making process is merely a suggestion to illustrate our point, based on our analysis of the literature.

**Figure 4:**
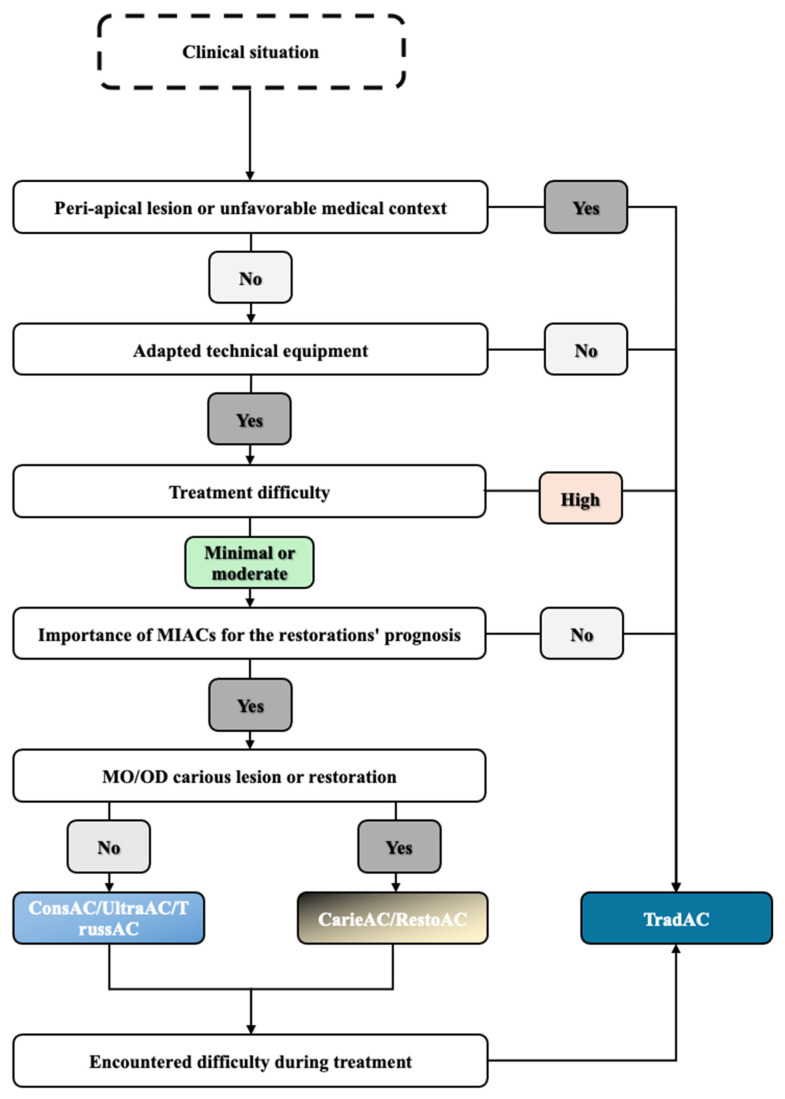
Synthesis and proposed decision tree for minimally invasive access cavities.

This diagram illustrates (Figure 4) the limited number of indications for MIACs and their numerous contraindications, which include the presence of a periapical lesion or unfavorable medical context, complex endodontic treatment, lack of appropriate technical equipment and lack of benefit to the tooth structure.

MIACs can be considered in limited indications, such as failed pulp capping procedures or cases of a necrotic tooth requiring endodontic treatment of moderate or low difficulty. However, advances in adhesive biomaterials and restorative techniques in endodontics may provide more effective solutions for maintaining the mechanical strength of treated teeth than reliance on MIACs designs. These innovations have the potential to achieve a favorable risk–benefit ratio without compromising the overall prognosis of endodontic therapy.

## 5. Conclusions

The results obtained are encouraging in terms of the mechanical properties of teeth treated with a conservative approach. However, this approach poses numerous challenges with regard to the endodontic treatment itself. It can compromise disinfection, lead to deviation from the original root canal path and affect the quality of obturation. In addition, these procedures appear to increase the likelihood of instrument deformation and fracture and prolong treatment time.

Such practices are relevant and recommended in certain limited cases; CariesACs and RestoACs may allow more conservative treatment where there is already substance loss. However, these new types of access cavities have many limitations and should only be used in cases with a favorable prognosis, minor difficulties and with adequate technical equipment.

Our conclusions are cautious due to the limited data available. The diversity of experimental parameters and the heterogeneity of protocols do not provide a high level of evidence on this topic. Future studies in this area should update our knowledge and further inform our practice.

## Figures and Tables

**Table 1:** Description of the 24 included studies with the seven relevant criteria.

Authors	Mechanical Resistance	Canal Orifice Detection	Instrumentation Quality	Canal Deviation	Instrument Constraints	Obturation	Time	Method
Abou-Elnaga 2019 [17]	X							TrussAC/TradAC, mandibular molars, *n* = 12
Al-Ani 2024 [18]			X					ConsAC/TradAC, mandibular and maxillary premolars/molars, *n* = 91, in vivo study
Alovisi 2018 [19]				X	X		X	ConsAC/TradAC, mandibular molars, *n* = 15
Barbosa 2020 [20]	X		X	X	X	X		TradAC/ConsAC/TrussAC, mandibular molars, *n* = 10
Chandolu 2024 [21]	X		X					TradAC/ConsAC, maxillary molar, *n* = 80
Corsentino 2018 [22]	X							TradAC/ConsAC/TrussAC, mandibular molars, *n* = 10
Kishan 2023 [23]				X				TradAC/TrussAC, mandibular molars, *n* = 15
Koohnavard 2023 [24]			X	X				TradAC/ConsAC, mandibular molars, *n* = 15
Krishan 2014 [25]	X		X	X				Trad/ConsAC, maxillary molars, premolars and incisors, n = 30
Kadhim 2023 [26]				X			X	TradAC/ConsAC, 2-rooted maxillary first premolars, *n* = 10
Lima 2021 [27]	X		X	X	X	X	X	TradAC/UltraAC, mandibular molars *n* = 10
Marchessan 2018 [28]				X	X		X	TradAC/ConsAC, mandibular molars, *n* = 12
Moore 2016 [14]	X		X		X			TradAC/ConsAC, maxillary molars, *n* = 12
Neelakan 2018 [29]			X					TradAC/TrussAC, mandibular molars, *n* = 12
Ozyürek 2018 [30]	X							TradAC/ConsAC, mandibular molars, *n* = 20
Pereira 2021 [31]	X		X	X		X		TradAC/ConsAC, maxillary premolars, *n* = 10
Plotino 2017 [32]	X							TradAC/ConsAC/UltraAC, maxillary and mandibular molars and premolars, *n* = 40
Rover 2017 [33]	X	X	X	X	X			TradAC/ConsAC, maxillary molars, *n* = 15
Rover 2020 [34]	X		X	X		X		TradAC/ConsAC (incisal edge), mandibular incisors, *n* = 10
Sabeti 2018 [35]	X							TradAC/ConsAC, maxillary molars, *n* = 16
Selvakumar 2023 [36]	X							TradAC/ConsAC, mandibular molars, *n* = 8
Silva 2020 [37]	X		X			X	X	TradAC/UltraAC, maxillary premolars, *n* = 10
Silva 2021 [38]					X			TradAC/UltraAC, mandibular molars, *n* = 10
Vieira 2020 [39]			X					TradAC/ConsAC, mandibular incisors, *n* = 30

## Abbreviations

The following abbreviations are used in this manuscript:

AAE	American Association of Endodontics.
CariesAC	Caries-driven access cavity.
ConsAC	Conservative access cavity.
EDTA	Ethylenediaminetetraacetic acid.
ESE	European Society of Endodontology.
MB2	Second mesio-buccal.
MIAC	Minimally invasive access cavity.
NaOCl	Sodium hypochlorite.
NinjaAC	Ninja access cavity.
RestoAC	Restorative-driven access cavity.
TradAC	Traditional access cavity.
TrussAC	Truss access cavity.
UltraAC	Ultra-conservative access cavity.
